# Identification of a prognostic six-immune-gene signature and a nomogram model for uveal melanoma

**DOI:** 10.1186/s12886-022-02723-1

**Published:** 2023-01-03

**Authors:** Binghua Yang, Yuxia Fan, Renlong Liang, Yi Wu, Aiping Gu

**Affiliations:** 1 Department of Ophthalmology, Guangdong Second Provincial General Hospital, Guangzhou, 510182 Guangdong China; 2 Department of Ophthalmology, Gansu Provincial Hospital, Lanzhou, 730000 Gansu China; 3 Department of Ophthalmology, Guangdong Second Provincial General Hospital, No. 466 Xin’gangzhong Road, Haizhu, 510317 Guangzhou China

**Keywords:** Uveal melanoma, Immune, Prognostic signature, Nomogram

## Abstract

**Background:**

To identify an immune-related prognostic signature and find potential therapeutic targets for uveal melanoma.

**Methods:**

The RNA-sequencing data obtained from The Cancer Genome Atlas (TCGA) and Gene Expression Omnibus (GEO) datasets. The prognostic six-immune-gene signature was constructed through least absolute shrinkage and selection operator and multi-variate Cox regression analyses. Functional enrichment analysis and single sample GSEA were carried out. In addition, a nomogram model established by integrating clinical variables and this signature risk score was also constructed and evaluated.

**Results:**

We obtained 130 prognostic immune genes, and six of them were selected to construct a prognostic signature in the TCGA uveal melanoma dataset. Patients were classified into high-risk and low-risk groups according to a median risk score of this signature. High-risk group patients had poorer overall survival in comparison to the patients in the low-risk group (*p* < 0.001). These findings were further validated in two external GEO datasets. A nomogram model proved to be a good classifier for uveal melanoma by combining this signature. Both functional enrichment analysis and single sample GSEA analysis verified that this signature was truly correlated with immune system. In addition, in vitro cell experiments results demonstrated the consistent trend of our computational findings.

**Conclusion:**

Our newly identified six-immune-gene signature and a nomogram model could be used as meaningful prognostic biomarkers, which might provide uveal melanoma patients with individualized clinical prognosis prediction and potential novel treatment targets.

**Supplementary Information:**

The online version contains supplementary material available at 10.1186/s12886-022-02723-1.

## Background

Uveal melanoma (UVM), the second most common form of melanoma, is a disease with highly aggressive features. UVM arises from melanocytes located in the uveal tract of the eye and it is the most common primary intraocular tumor [1]. Its incidence is often less than 10 per million population one year [2]. The main treatment options, including enucleation, local resection, and radiation therapies, are recommended by the National Comprehensive Cancer Network (NCCN) guideline [3]. However, the mortality rate is still rather high over the past decades [4]. In particular, about half of diagnostic UVM patients may have distant metastasis via hematogenous spreading, such as liver, which has a mean overall survival time of not more than one year [5]. Therefore, it is of great urgency to find novel useful biomarkers to predict the prognosis of UVM patients.

UVM develops at an immune-privileged site, and immunotherapy may not be sufficient to achieve satisfactory therapeutic effects on UVM as previously reported [2, 6]. However, encouraging discoveries about immune therapy on UVM have been reported recently. Tebentafusp, an immune melanoma-associated-antigen (gp100)-targeting anti-CD3 / T-cell receptors (TCRs) bispecific fusion protein, has been designed to guide T lymphocytes to kill gp100-expressing UVM cells, and Tebentafusp results in a longer overall survival than the control therapy among previously untreated patients with metastatic uveal melanoma [7]. In 2020, a multicenter phase I/II clinical trial about Tebentafusp conducted on eighty-four metastatic UVM patients reported a promising one-year overall survival rate of 65% [8], which was consistent with previous reports [9]. In addition, a combination treatment of anti-angiogenic therapy with immunotherapy to cure metastatic UVM was proposed, based on the theory that targeting vascular endothelial growth factor (VEGF) could not only inhibit angiogenesis, but also change the tumor microenvironment, which would make UVM cells more immune-responsive [10]. Meanwhile, this theory has been successfully proved in some malignant tumors, such as advanced cutaneous melanoma [11], non-small cell lung cancer [12], and advanced renal cell carcinoma [13].

Taken together, immunotherapy might demonstrate a bright future in treating UVM patients. However, few studies have systematically studied useful immune biomarkers to predict an overall survival outcome for UVM patients and find out UVM patients who might benefit from immunotherapy option. Therefore, in the current study, we assumed that a comprehensive immune-gene signature can be a prognostic biomarker for UVM patients. Hence, univariate and multivariable Cox regression analysis was conducted, followed by LASSO regression analysis, to select immune- and prognosis- related genes. Then the prognostic efficacy of the genes was evaluated using the datasets from the Cancer Genome Atlas (TCGA) UVM cohort, and validated using two datasets from the Gene Expression Omnibus (GEO). A prognostic nomogram model was constructed to provide some helpful information for UVM patients prognosis and therapy. Besides, the role of *CCL18* in the UVM cell phenotype was finally verified by cytological experiments.

## Materials and methods

### The collection of RNA-sequencing data with clinical information

The transcriptome profiling datasets with clinical features were downloaded from the TCGA (https://portal.gdc.cancer.gov/; Project ID: TCGA-UVM). The microarray datasets with clinical information were downloaded from the GEO (https://www.ncbi.nlm.nih.gov/gds; GEO Accession: GSE84976 and GSE22138).

### Construction of prognostic scoring model based on immune genes in the TCGA-UVM dataset

Three hundred thirty-two immune genes were summarized from two immune-related gene sets in the Molecular Signatures Database v4.0 ( http:// www. broadinstitute. org / gsea / msigdb / index. jsp; IMMUNE_SYSTEM_PROCESS: M13664, IMMUNE_RESPONSE: M19817). The “edgeR” package in R language (version 4.0.3) was used to select the differentially expressed genes (DEGs). Univariate Cox regression and K-M survival analyses were performed to identify the genes associated with prognosis using survival package in R language. The optimal panel of prognostic immune genes were selected based on the least absolute shrinkage and selection operator (LASSO) regression analysis. LASSO analysis was carried out using glmnet package in R language. The penalization coefficient lambda was obtained by running cross-validation deviance (nfolds = 10). “lambda.1se” was chosen as the optimal lambda. The risk score formula was as following: Risk score = Exp_immune_gene-1 × Coef_immune_gene-1 + Exp_immune_gene-2 × Coef_immune_gene-2 + … + Exp_immune_gene-n × Coef_immune_gene-n. Where Exp means the expression level of the immune genes, and Coef is Coefficient.

### Evaluation of the prognostic six-immune-gene signature in the TCGA UVM dataset and validation in two GEO datasets

Based on this newly identified six-immune-gene signature, individual patients were divided into the high- or low-risk group using a median cutoff value in the TCGA UVM dataset. Further analyses of survival time, status of each patient (dead or alive) and heatmap of 6 genes expression (*PRELID1*, *JAG2*, *GTPBP1*, *PTGER4*, *CCL18*, and *CXCL8*) were performed. The distributions of risk score and survival status were plotted for UVM patients. The heatmap package was used to acquire the gene expression profile of 6 immune-related genes in UVM patients. The K-M survival analysis was performed and visualized using survminer and survival packages in R language. Time-dependent ROC (1-year, 2-year, and 3-year) and multivariate ROC analyses were conducted to evaluate the diagnostic efficacy by using the time ROC, survival ROC, survminer, and survival packages in R language. Principal components analysis of the six-immune-related gene signature was visualized for the high-risk and low-risk groups. In addition, the univariate and multivariate Cox regression analyses were utilized to evaluate the efficiency of this signature to independently predict the survival outcomes of UVM patients. UVM patients in the GSE84976 and GSE22138 were grouped into the high- or low-risk groups using a median cutoff value in the TCGA UVM dataset, which were used for evaluation of both stability and reliability of the model.

### Clinical correlation and stratification survival analysis of the prognostic six-immune-gene signature in the TCGA UVM dataset

To further evaluate the potential clinical application of this signature, the clinicopathological variables in the TCGA UVM dataset were stratified into different subgroups accordingly. This includes age (< 65 and ≥ 65), gender (male and female), histological subtype (single spindle cell (E) / epithelioid cell (S)) and mixed subtype), TNM stage (stage II + III and stage IV), T stage (T 2 + 3 and T 4), M stage (M0 and M1), N stage (N0 and N1), new tumor event (NO and YES), and tumor basal diameter (< 15 and ≥ 15). The risk score values were compared between different subgroups by using beeswarm package in R language. The K-M survival analysis was performed by using the survminer and survival packages in R language.

### Functional enrichment analysis in the TCGA UVM dataset

To further reveal the potential enriched pathway functions between high- and low-risk group patients based on this newly identified signature, the cluster Profiler, org.HS.eg.dbm enrichplot, and ggplot2 packages in R language was used to conduct the Gene Ontology (GO) [14] and Kyoto Encyclopedia of Genes and Genomes (KEGG) [15–17] enrichment analyses (*p*-value < 0.05).

### Single sample Gene Set Enrichment Analysis (ssGSEA) in the TCGA UVM dataset

Single sample Gene Set Enrichment Analysis (ssGSEA), as an extensive application of GSEA, it calculates an enrichment score, which represents that the genes in a particular gene set is increased or decreased in a sample. The ssGSEA was used to quantify the enrichment levels of immune cells, and immune functions between high risk and low risk groups patients. ssGSEA was carried out using GSEA software (https://www.gsea-msigdb.org/gsea/index.jsp). The enrichment scores more than 0.4 and FDR values less than 0.05 indicate a statistical significance.

### Construction and validation of a predictive nomogram model in the TCGA UVM dataset

Multivariate Cox proportional hazards regression analysis was carried out to screen variables affecting overall survival in the TCGA UVM dataset. A predictive nomogram model was constructed by combining the age, gender, histological type, TNM stage, new tumor event, tumor basal diameter, and risk scores. A nomogram for predicting 1-, 2-, and 3- year was constructed. The concordance index (C-index) and the area under the ROC (AUC) were used to evaluate the discriminative ability of the constructed nomogram based on the training set. The calibration analysis was performed to explore the discrimination by using a bootstrap method. The patients were separated into low- and high-nomogram-score groups by the median cutoff value of nomogram score, and K-M survival was analyzed to evaluate the diagnostic efficacy. Nomogram models were constructed using the rms package in R language. The sensitivity and specificity were calculated using the survival ROC package in R language. C-index was calculated using the survival package in R language.

### Cell culture and transfection

Human uveal melanoma cell lines Um95, M17, M23, and SP6.5 cells were obtained from the Cell Bank of Chinese Academy of Sciences (Shanghai, China) in this study. Human uveal melanocytes Um95 and M17 were originally isolated according to previous method [18]. Melanoma cell line SP6.5 were derived from primary tumors of the patients confirmed with UVM [19]. Uveal melanoma cell line B M17 was originally isolated from choroidal melanoma patients [20]. Um95, M23, M17 and SP6.5 cells were maintained in complete DMEM medium containing 10% FBS (GE™ Hyclone, Utah, U.S.), 100 U/ml penicillin and 100 mg/ml streptomycin (SH30010, GE™ Hyclone, Utah, U.S.). Um95, M23, M17, and SP6.5 cells were cultured in a humidified atmosphere of 37 °C and 5% CO_2_. The cells were sub-cultured one day before transfection to achieve a confluency of 30%-50%.

Lipofectamine 2000 was used for transfection with a working concentration of 50 nM and OPTI-MEM medium was used for transfection. Small interfering RNA targeting CCL18 was purchased from Thermo Fisher Scientific (Waltham, MA, USA). siRNA sequences are as follows, siRNA-1 (5’-GUU CAU AGU UGA CUA UUC UUU-3’) and siRNA-2 (5’-GUG CAC AAG UUG GUA CCA AUU-3’). After incubation for 4 h, the cells were replaced with cell growth medium. Cell functional cell proliferation was detected 48 h later.

### Realtime PCR

The relative expression of CCL18 mRNA in Um95, M23, M17 and SP6.5 cells was detected by realtime PCR. Whole cell RNA was extracted by Trizol (15,596,018, Life Technologies, USA) method and then RNA transcribed into cDNA using a BeyoRT™ III cDNA Synthesis Kit (Beyotime Biotechnology, Shanghai, China). BeyoFast™ SYBR Green qPCR Mix (2X) (D7260, Beyotime Biotechnology, Shanghai, China) kit were used to performed Real-time PCR assay. The qPCR experimental results were calculated by 2^−△△CT^ method. Primer sequences were shown in Table 1.

**Table 1:** Primer sequences for qPCR

**Gene**	**Primer sequences**
CCL18	F: GTTGACTATTCTGAAACCAGCCC
	R: GTCGCTGATGTATTTCTGGACCC
CXCL8	F: GAGAGTGATTGAGAGTGGACCAC
	R: CACAACCCTCTGCACCCAGTTT
GTPBP1	F: CCTTCATCGACTTGGCTGGTCA
	R: CCAGGTGTTCTTTGGTCATCCC
JAG2	F: GCTGCTACGACCTGGTCAATGA
	R: AGGTGTAGGCATCGCACTGGAA
PRELID1	F: GGAGGACTCTATTGTGGACCCA
	R: CAGTCCAGCCACTGTTGTCAGA
PTGER4	F: TACTCATTGCCACCTCCCTGGT
	R: GACTTCTCGCTCCAAACTTGGC
GAPDH	F: GTCTCCTCTGACTTCAACAGCG
	R: ACCACCCTGTTGCTGTAGCCAA

### Western Blot

We used BeyoLytic™ Mammalian active protein extraction reagents (Beyotime Biotechnology, Shanghai, China) to extract the total cell protein. Then 30–40 μg total cell protein were used to perform SDS–polyacrylamide gel electrophoresis and protein transfer assay. Then the transferred NC membrane incubated with the corresponding primary antibody: Anti-CCL18 (ab233099, abcam, USA) and Anti-GAPDH (ab8245, abcam, USA) (1:500) at 4 °C overnight.

### CCK8 counting assay

Cell proliferation was examined using the cell counting kit-8 (CCK-8; Dojindo, Kumamoto, Japan). Cells were seeded into 96-well plates. Here, 1 × 10^4^ cells per well were planted. The cells were collected 0 h, 24 h, 48 h, and 72 h after incubation. Then the culture was added with 10 ul of CCK8 solution single solution cell proliferation detection liquid. After incubating for 4 h, the absorbance of 450 nm was detected with a microplate reader.

### Annexin V-FITC Apoptosis Detection

We used Annexin V-FITC Apoptosis Detection Kit (C1062S, Beyotime Biotechnology, Shanghai, China) to detect cell apoptosis, and carried out experimental operations according to Annexin V-FITC Apoptosis Detection Kit 's instructions.

### Cell migration and invasion assay

IN this study, we performed trans-well assay to detect cell migration and invasion followed the “In vitro Cell Migration and Invasion Assays, https://doi.org/10.3791/51046” article instructions.

### Statistical analysis

The data of our study were extracted and sorted using the PERL programming language (http://www.perl.org/, Version 5.30.0). Gene expression data in TCGA-UVM were extracted using the json package of perl software. The clinical data files and pathological data were analyzed using the XML::Simple package. All statistical analyses and data visualization were carried out by using the R software (v 4.0.3: http://www.r-project.org). The data used in this paper are expressed as mean ± standard error of three independent measurements. All statistics were analyzed by Student’s t-test, and the data analysis software was GraphPad Prism 6. *P* < 0.05 was considered statistically significant. *P < 0.05; ** *P* < 0.01; ***P < 0.001.

## Results

### Clinical characteristics of all UVM patients

The TCGA UVM dataset (*N* = 80) was utilized to construct the prognostic immune-gene signature. GSE84976 (*N* = 28) and GSE22138 (*N* = 63) datasets were used as the validation cohorts. All clinical characteristics are summarized in Table 2.

**Table 2:** Clinical characteristics of included uveal melanoma patients in the TCGA dataset and two GEO datasets (GSE84976 and GSE22138). Mixed: Spindle Cell | Epithelioid Cell, and Epithelioid Cell | Spindle Cell

Characteristics	TCGA UVM (*N* = 80)	GSE84976 (*N* = 28)	GSE22138 (*N* = 63)
Age at diagnosis (years)			
< 65	45 (56.25%)	12 (42.86%)	36 (57.14%)
≥ 65	35 (43.75%)	16 (57.14%)	27 (42.86%)
Sex			
Male	45 (56.25%)	-	40 (63.49%)
Female	35 (43.75%)	-	23 (36.51%)
Histological subtype			
Spindle Cell	30 (37.50%)	-	-
Epithelioid Cell	13 (16.25%)	-	21 (33.33%)
Mixed	37 (46.25%)	-	23 (36.51%)
NA	-	-	19 (30.16%))
TNM stage			
Stage I	0 (0.00%)	-	-
Stage II	36 (45.00%)	-	-
Stage III	40 (50.00%)	-	-
Stage IV	4 (5.00%)	-	-
New tumor event			
NO	60 (75.00%)	-	-
YES	20 (25.00%)	-	-
Tumor basal diameter			
< 15	20 (25.00%)	-	-
≥ 15	60 (75.00%)	-	-

### Identification of the prognostic six-immune-gene signature in TCGA UVM dataset

**Fig. 1:**
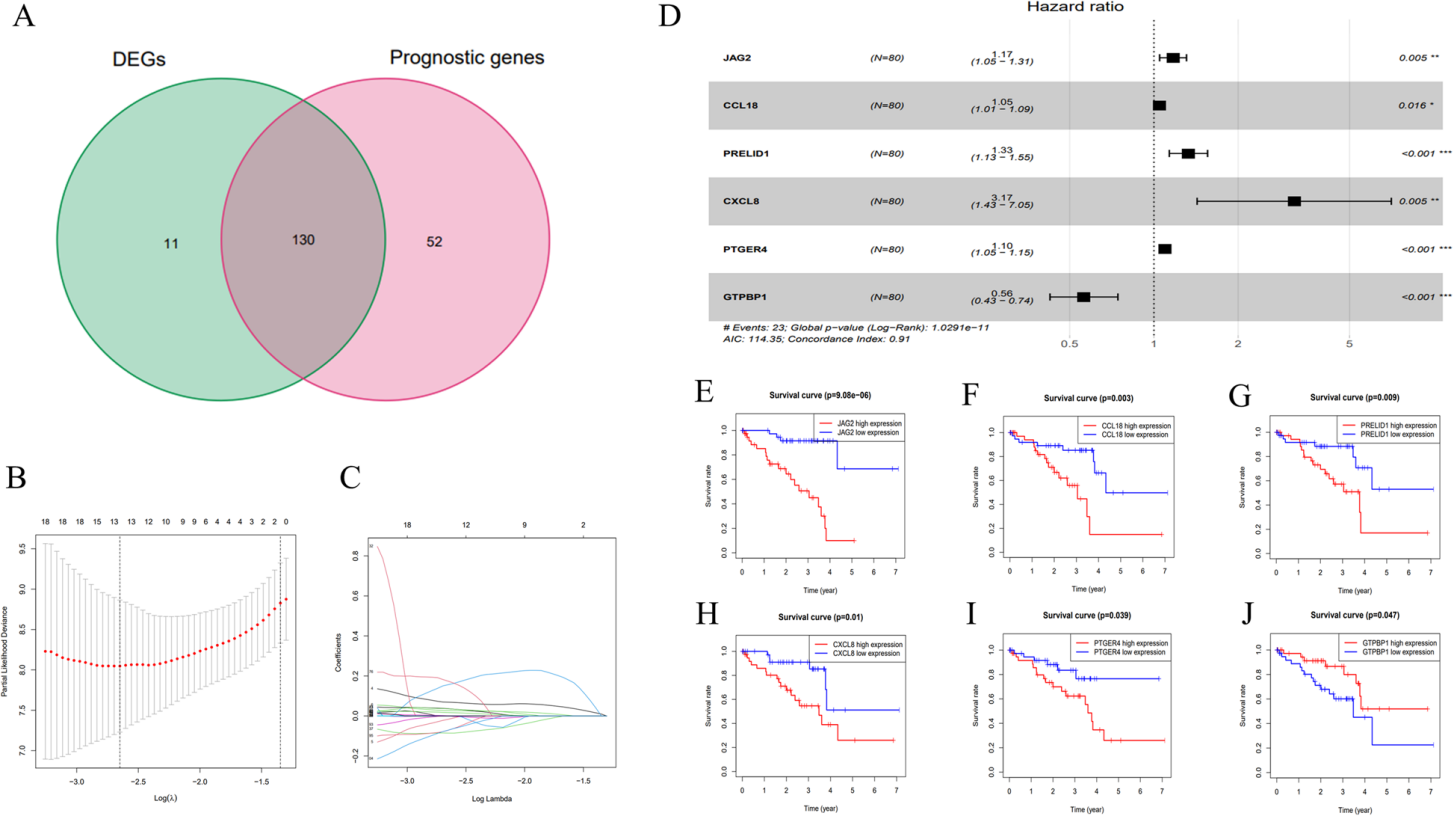
Identification of the six-immune-related gene signature in TCGA UVM dataset. **A** Venn plot of overlapping results of univariate Cox regression analysis and KM survival analysis; (**B**) Cvfit plot of LASSO cox regression analysis; (**C**) Lambda plot of LASSO Cox regression analysis; (**D**) Hazard ratio of the six-immune-related gene signature in multivariate Cox regression analysis; K-M survival analysis of the six selective immune-related genes: (**E**) JAG2; (**F**) CCL18; (**G**) PRELID1; (**H**) CXCL8; (**I**) PTGER4; (**J**) GTPBP1

A total of 332 immune-related genes were obtained, and 141 genes were selected considering the differentially expressed level compared to control cohorts (Fig. 1A). After univariate Cox regression and K-M survival analysis, 130 immune genes associated with prognosis were selected. Tunning parameter (*λ*) was calculated by the LASSO regression based on tenfold cross-validation (Fig. 1B). The most appropriate *λ* for LASSO regression was ensured. Then, 14 prognostic immune genes with nonzero coefficients were selected by LASSO Cox regression analysis (Fig. 1C). Multivariable Cox regression analysis was performed, and finally a six-immune-related-gene signature was identified, including *JAG2*, *CCL18*, *PRELID1*, *CXCL8*, *PTGER4*, and *GTPBP1* (Fig. 1D), whose risk score of each patient was generated using the following risk score formula: Risk score = Exp_JAG2 × 0.157 + Exp_ CCL18 × 0.046 + Exp_ PRELID1 × 0.284 + Exp_ CXCL8 × 1.154 + Exp_ PTGER4 × 0.092- Exp_ GTPBP1 × 0.576 (Table 3). In addition, the K-M survival results of these six immune genes were presented in Fig. 1E-J, suggesting the expression level of these 6 immune-related genes was greately associated with the survival rate of UVM patients.

**Table 3:** The six-immune-related signature identified from multivariate Cox analysis

			Multivariate Cox regression analysis		
Gene symbol	Description	Coefficient	HR	95%CI	*p*-value
JAG2	Jagged2	0.157	1.170	1.048–1.307	0.005
CCL18	Chemokine 18	0.046	1.047	1.008–1.087	0.016
PRELID1	PRELI domain-containing protein 1	0.284	1.328	1.135–1.555	< 0.001
CXCL8	Interleukin 8	1.154	3.172	1.427–7.053	0.005
PTGER4	Prostaglandin E (2) receptor	0.092	1.096	1.047–1.148	< 0.001
GTPBP1	GTP-binding protein 1	-0.576	0.562	0.425–0.743	< 0.001

### Evaluation of the six-immune-gene signature in TCGA UVM dataset

**Fig. 2:**
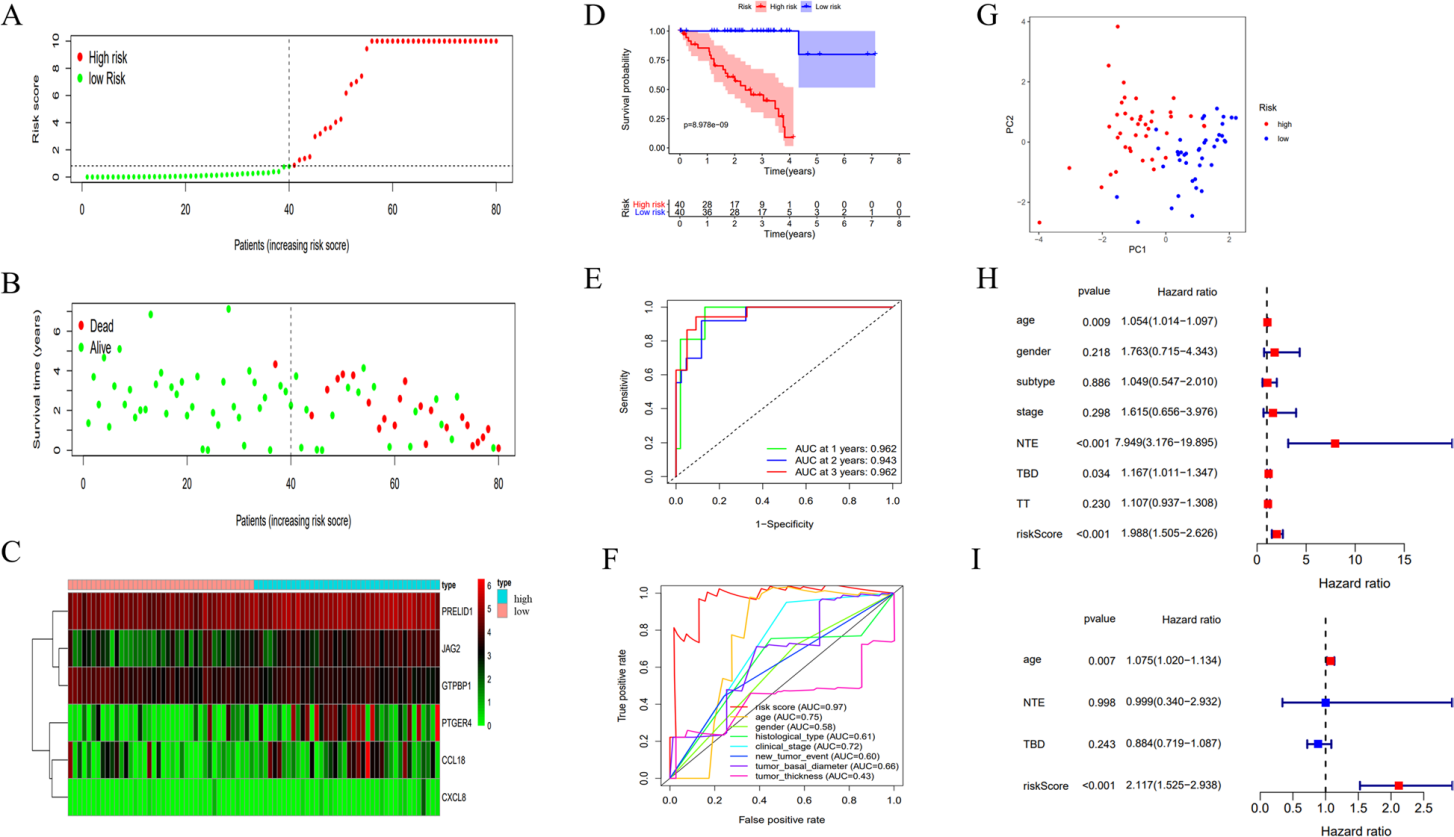
Evaluation of the six-immune-related gene signature in TCGA UVM dataset. **A**-**C** Distribution of risk score, survival status, and expression heatmap of each patient; (**D**) K-M survival curve of the high-risk and low-risk groups; (**E**) The time-dependent receiver operating characteristic (ROC) curves and area under the curve (AUC) at 1-, 2-, and 3 years; (**F**) ROC curve analysis showed the prognostic accuracy of clinicopathological parameters and the signature risk score; (**G**) Principal components analysis (PCA) of the six-immune-related gene signature; (**H**) Forest plot for univariate Cox regression analysis; (**I**) Forest plot for multivariate Cox regression analysis

The risk score, survival time and survival status of each patient are plotted in Fig. 2A-B. The heatmap is displayed in Fig. 2C. The overall survival time of the low-risk group patients was significantly higher than that of the high-risk group patients (Fig. 2D, *p* < 0.001). The AUCs of the time-dependent ROC at 1-, 2- and 3- year were 0.962, 0.943, and 0.962, respectively (Fig. 2E). The AUC of multi-variate ROC for the 6-immune-gene signature risk score was 0.97, which was much better than that of other clinical variables (Fig. 2F). The PCA analysis showed that this six-immune-gene signature could help distinguish the high-risk patients from the low-risk patients ideally (Fig. 2G).

Moreover, the results of univariate and multivariate Cox regression analyses of the age, gender, histological type, TNM stage, new tumor event, tumor basal diameter and this signature risk score, are shown in Fig. 2H-I. Risk score of this newly identified signature (HR = 2.117, 95%CI = 1.525–2.938, *p* < 0.001) was an independent clinical prognostic risk factor (Fig. 2I).

### Validation of the six-immune-gene signature in the two GEO datasets

**Fig. 3:**
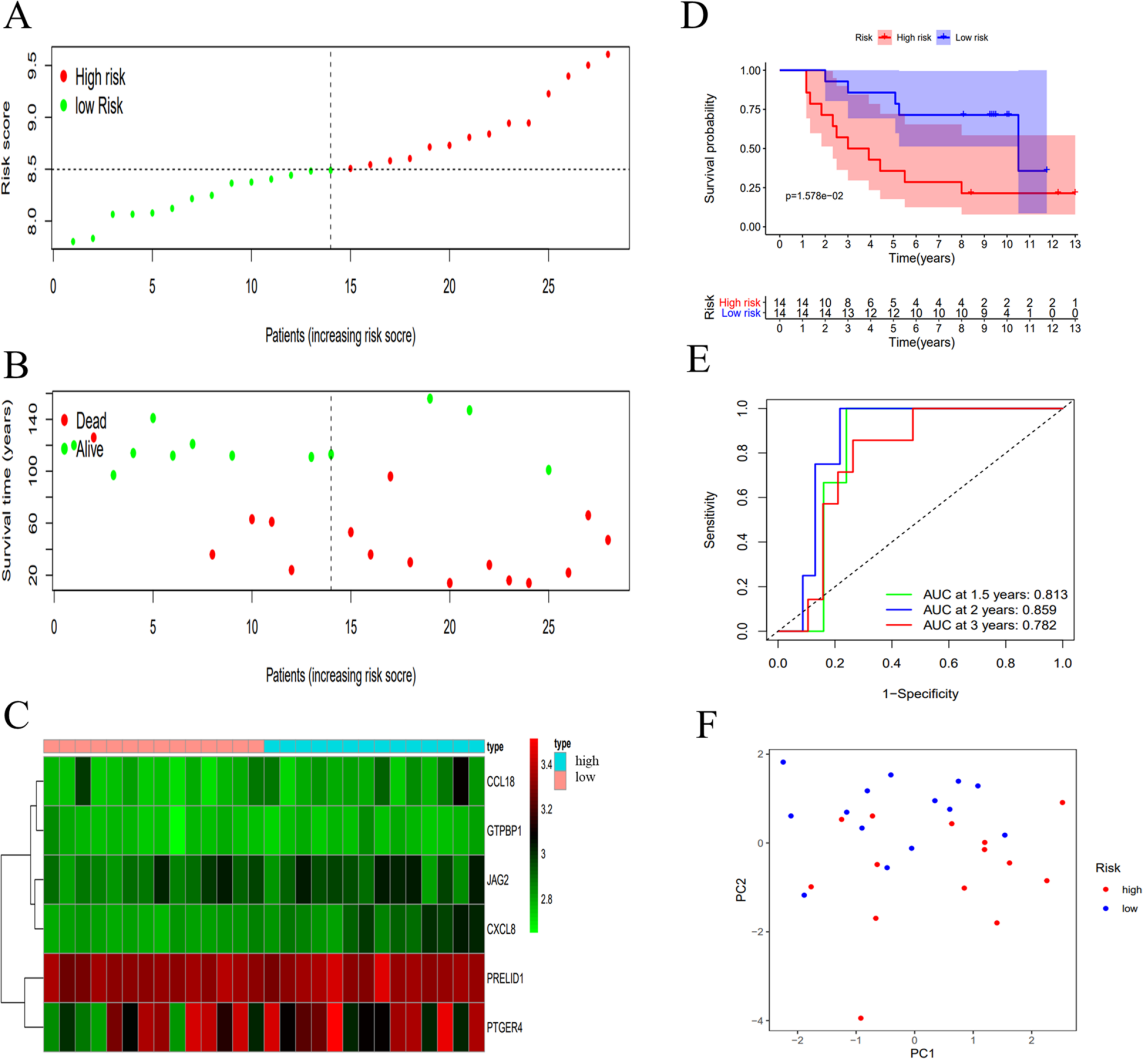
Validation of the six-immune-related gene signature in GSE84976 dataset. **A**-**C** Distribution of risk score, survival status, and expression of each patient; (**D**) K-M survival curve of the high-risk and low-risk groups patients; (**E**) Time-dependent ROC curves and AUC at 1-, 2-, and 3 years; (**F**) PCA of the six-immune-related gene signature

**Fig. 4:**
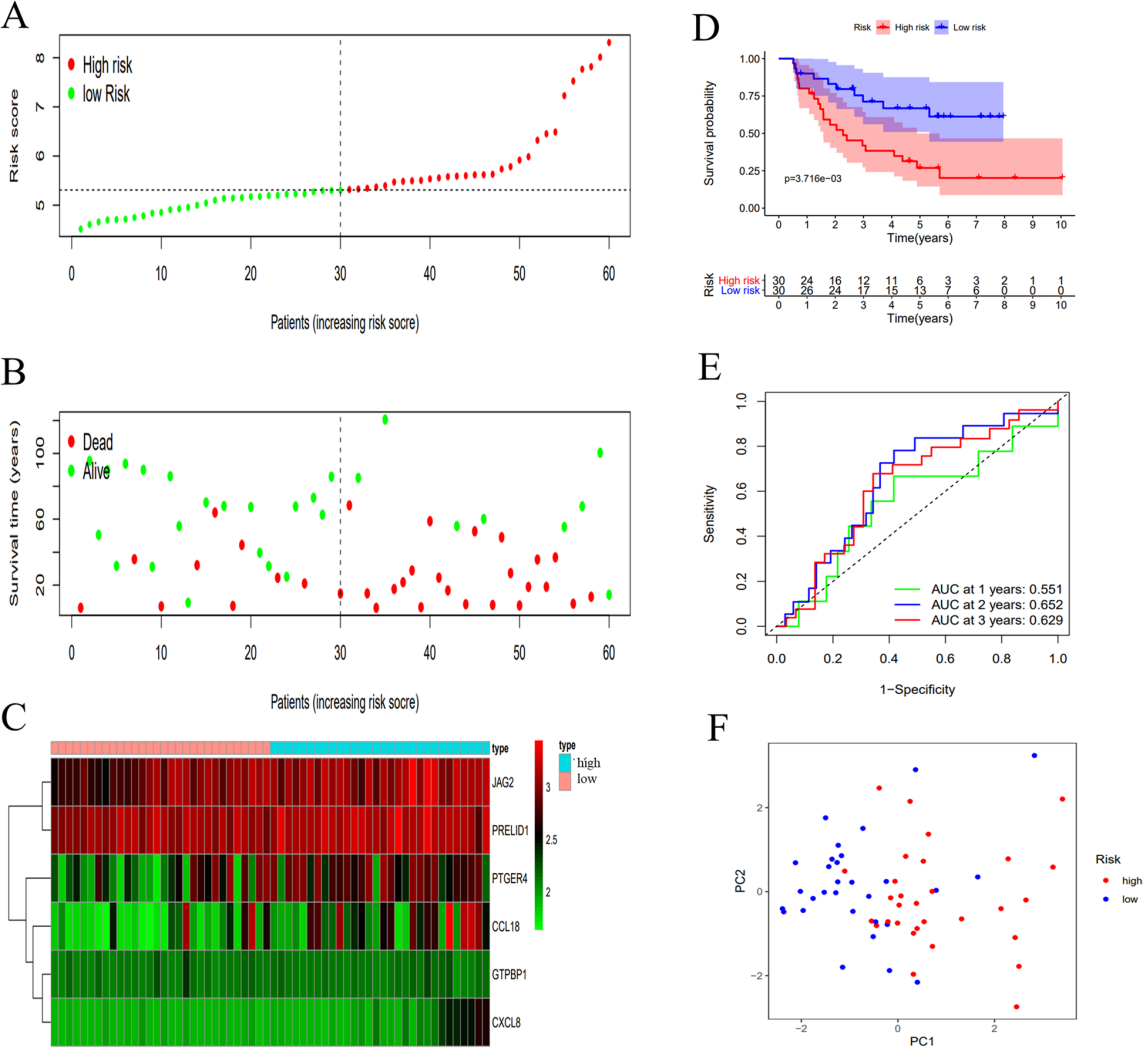
Validation of the six-immune-related gene signature in GSE22138 dataset. **A**-**C** Distribution of risk score, survival status, and expression of each patient; (**D**) K-M survival curve of the high-risk and low-risk groups patients; (**E**) Time-dependent ROC curves and AUC at 1-, 2-, and 3 years; (**F**) PCA of the six-immune-related gene signature

To further confirm the predictive diagnostic power and stability of this six-immune-gene signature in predicting the overall survivals of UVM patients, we validated it in two GEO datasets, including GSE84976 (*N* = 28) (Fig. 3) and GSE22138 (*N* = 63) (Fig. 4). Risk scores were also generated using the same risk score formula constructed in the TCGA UVM dataset.

The risk score, survival time and survival status of each patient in the two GEO datasets were displayed (Fig. 3A-B, 4A-B). The heat map of the expression of this six-immune-gene signature was plotted (Fig. 3C, 4C). The overall survival time of the low-risk group patients was significantly higher than that of the high-risk group patients (Fig. 3D, 4D, *p* < 0.001). In addition, in GSE84976, the AUCs of the time-dependent ROC at 1-, 2- and 3- year were 0.813, 0.859, and 0.782, respectively (Fig. 3E). In GSE22138, the AUCs of the time-dependent ROC at 1-, 2- and 3- year were 0.551, 0.652, and 0.629, respectively (Fig. 4E). The PCA analysis indicated that this six-immune-gene signature could largely help distinguish the high-risk patients from the low-risk patients (Fig. 3F, 4F).

### Clinical correlations in the TCGA UVM dataset

**Fig. 5:**
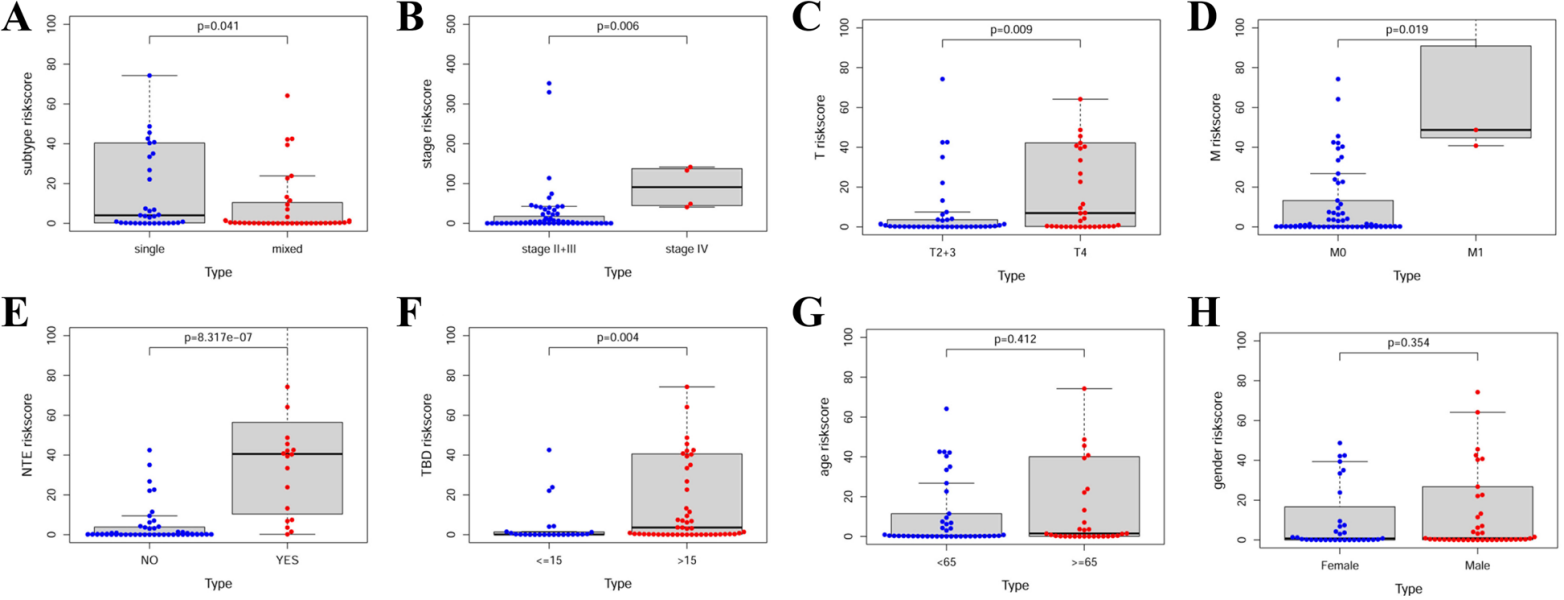
Clinical correlations in the TCGA UVM dataset. The risk score distributions between the signature risk scores and the clinicopathological features in different subgroups: (**A**) histological subtypes (single vs mixed subtype; *p* = 0.041) (single: spindle cell subtype and epithelioid cell subtype; mixed: Epithelioid Cell | Spindle Cell, and Spindle Cell | Epithelioid Cell); (**B**) TNM stage (stage II + III vs stage IV; *p* = 0.006); (**C**) T stage (T 2 + 3 vs T 4; *p* = 0.009); (**D**) M stage (M0 vs M1; *p* = 0.019); (**E**) new tumor event (NTE, NO vs YES, *p* = 8.317e-07); (**F**) tumor basal diameter (TBD, ≥ 15 vs < 15, *p* = 0.004); (**G**) age (≥ 65 vs < 65; *p* = 0.412); (**H**) gender (female vs male; *p* = 0.354)

Consistent with our expectation, patients with single subtype, higher TNM stages (stage IV), higher T stage (T4), higher M stage (M1), new tumor event (YES), and tumor basal diameter (≥ 15), had significant higher risk scores than those with mixed subtype, lower TNM stages (stage II + III), lower T stage (T2 + 3), M0, new tumor event (NO), and tumor basal diameter (< 15) (all *p* < 0.05) (Fig. 5A-F). The risk score in age (Fig. 5G) and gender (Fig. 5H) did not show significant differences. These findings suggested that high risk score of this signature might be involved with the disease progression of UVM patients.

### Stratification survival analysis in the TCGA UVM dataset

**Fig. 6:**
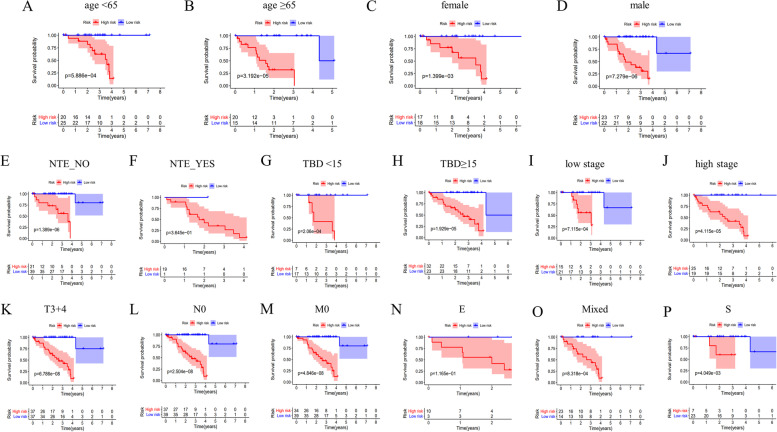
Stratification survival analysis in the TCGA UVM dataset. **K**-**M** survival analysis showed the overall survival time of the high- and low-risk UVM patients stratified by different variables: age (**A**-**B**); sex (**C**-**D**); new tumor event (NTE, **E**–**F**); tumor basal diameter (TBD, **G**-**H**); TNM stage (**I**-**J**), T stage (**K**), N stage (**L**), M stage (**M**), E: epithelioid cell subtype (**N**); Mixed: Epithelioid Cell | Spindle Cell, and Spindle Cell Epithelioid Cell (**O**); S: spindle cell subtype (**P**)

Compared with the patients in the low-risk group, those in the high-risk group had a worse outcome in many different subgroups, including age (Fig. 6A-B), gender (Fig. 6C-D), new tumor event (NO) (Fig. 6E), tumor basal diameter (Fig. 6G-H), TNM stage (Fig. 6I-J), T3 + 4 (Fig. 6K), M0 (Fig. 6L), N0 (Fig. 6M), mixed subtype (Fig. 6O), and spindle cell subtype (Fig. 6P) (all *p* < 0.05), but not in the group with new tumor event (YES) (Fig. 6F), and epithelioid cell subtype (Fig. 6N) (all *p* > 0.05). However, they all showed the same tendency.

### Functional enrichment analysis in the TCGA UVM dataset

**Fig. 7:**
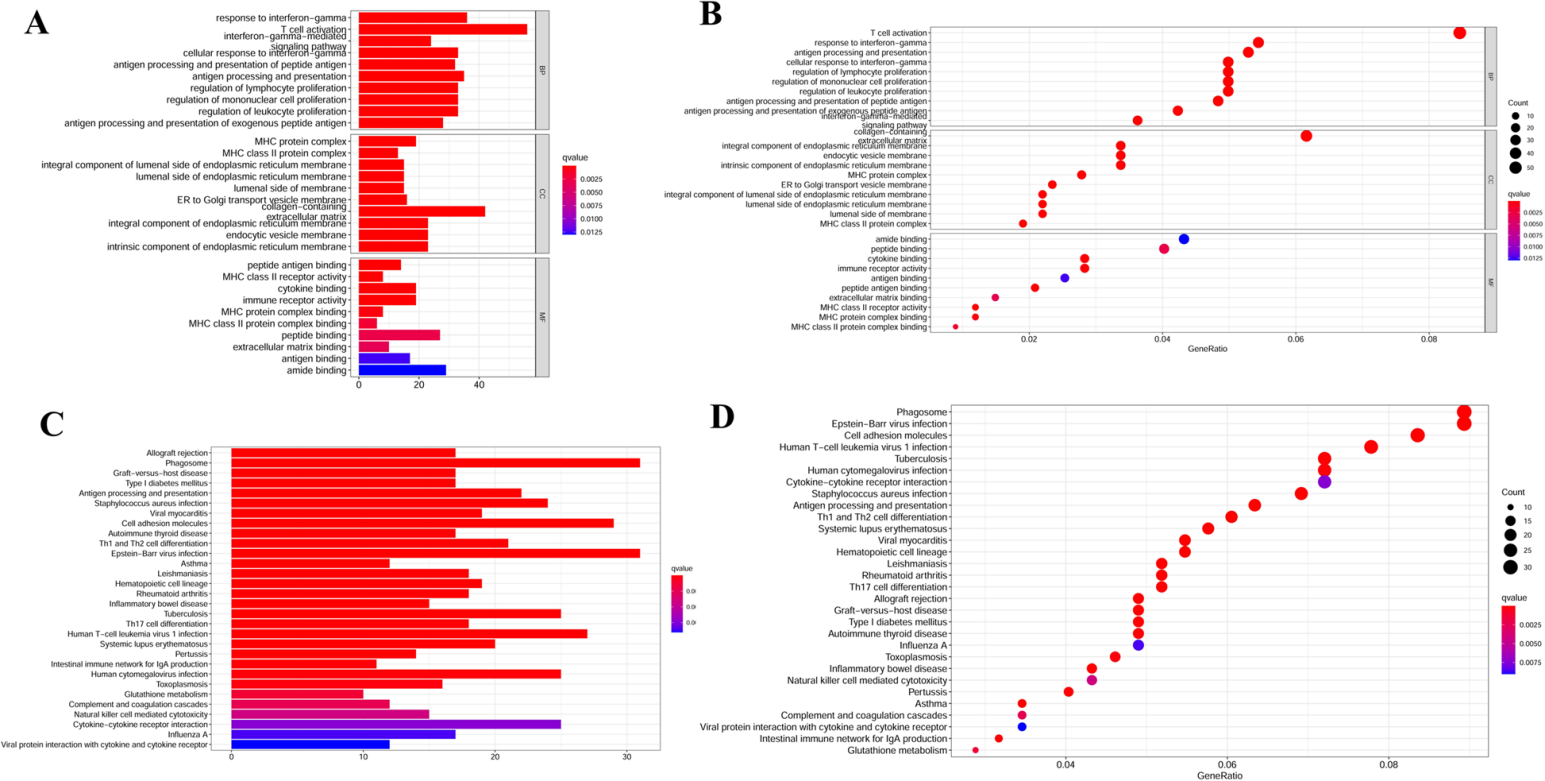
Functional enrichment analysis of six-immune-related gene signature in the TCGA UVM dataset. The bar plot and dot plot of Gene Ontology (GO) (**A**-**B**) and Kyoto Encyclopedia of Genes and Genomes (KEGG) pathway (**C**-**D**) functional enrichment analyses. BP: Biological process; CC: Cellular Component; MF: Molecular function

The functional enrichment analysis of GO enrichment categories, including biological process (BP), cell component (CC) and molecular function (MF), displayed the enrichment of some known immune-related pathways, including response to interferon gamma, T cell activation, interferon-gamma-mediated signaling pathway, cellular response to interferon-gamma, antigen processing and presentation of peptide antigen, and so on (Fig. 7A-B). Similar result was also obtained from KEGG pathway enrichment analysis (Fig. 7C-D).

### ssGSEA analysis in the TCGA UVM dataset

**Fig. 8:**
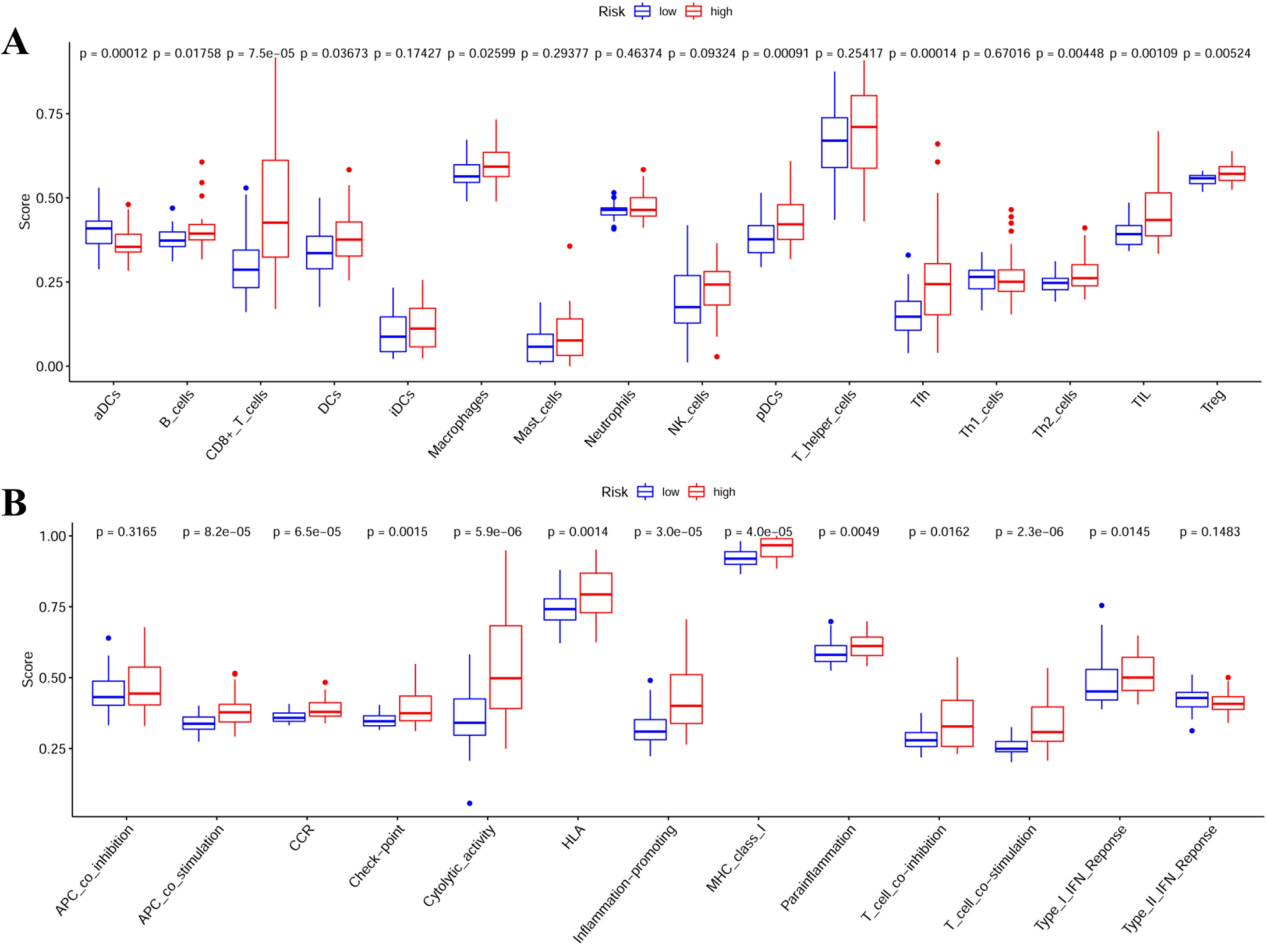
Single-sample GSEA (ssGSEA) in the TCGA UVM dataset. **A** The enrichment score of immune cells in the high-risk and low-risk group patients in ssGSEA; (**B**) The enrichment score of immune functions in the high-risk and low-risk group patients in ssGSEA

The ssGSEA indicated that the high risk patients were enriched of many immune cells, including B cells, CD8 + T cells, DCs, macrophages, pDCs, Tfh, Th2 cells, TIL, and Treg, while the low risk patients were only enriched in aDCs (Fig. 8A, all *p* < 0.05). High risk patients are enriched in all immune functions (all *p* < 0.05), except for APC-co-inhibition and Type-II IFN response (Fig. 8B, all *p* > 0.05).

### Construction and evaluation of the predictive nomogram model in the TCGA UVM dataset

**Fig. 9:**
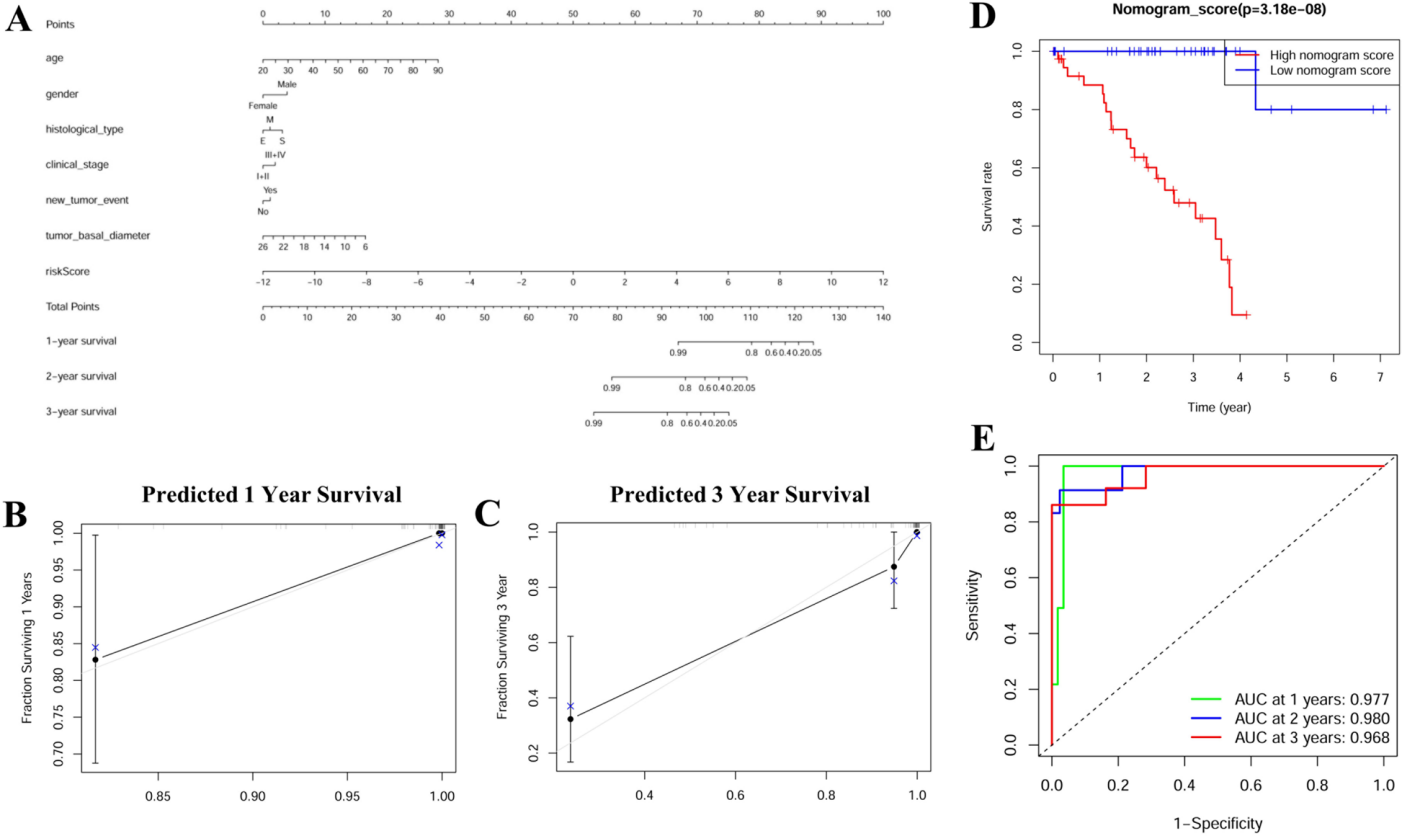
Construction and evaluation of the prognostic nomogram model in the UVM dataset. **A** The nomogram model was constructed by age, gender, histological type, TMN stage, new tumor event, tumor basal diameter and six-immune-related prognostic signature risk score; (**B**-**C**) The calibration plot of the nomogram; (**D**) K-M survival curve between high-nomogram-score and low-nomogram-score groups; (**E**) The AUCs of the time-dependent ROC curves at 1-, 2-, and 3 years

This newly identified predictive nomogram model was successfully constructed by combining all the details of age, gender, histological type, TNM stage, new tumor event, tumor basal diameter and this signature risk score in TCGA UVM dataset (Fig. 9A). The calibration plots suggested that no significant deviations between the observed and predicted curves were found for both 1-year and 3-year survivals (Fig. 9B-C). The UVM patients in high-nomogram-score group had a worse outcome compared with those in the low nomogram-score group (*p* < 0.001, Fig. 9D). The AUCs of time-dependent ROC curves for 1-, 2- and 3 years were 0.977, 0.980, and 0.968, respectively (Fig. 9E).

### Knocking-down of CCL18 expression inhibits uveal melanoma cells proliferation, migration and invasion

**Fig. 10:**
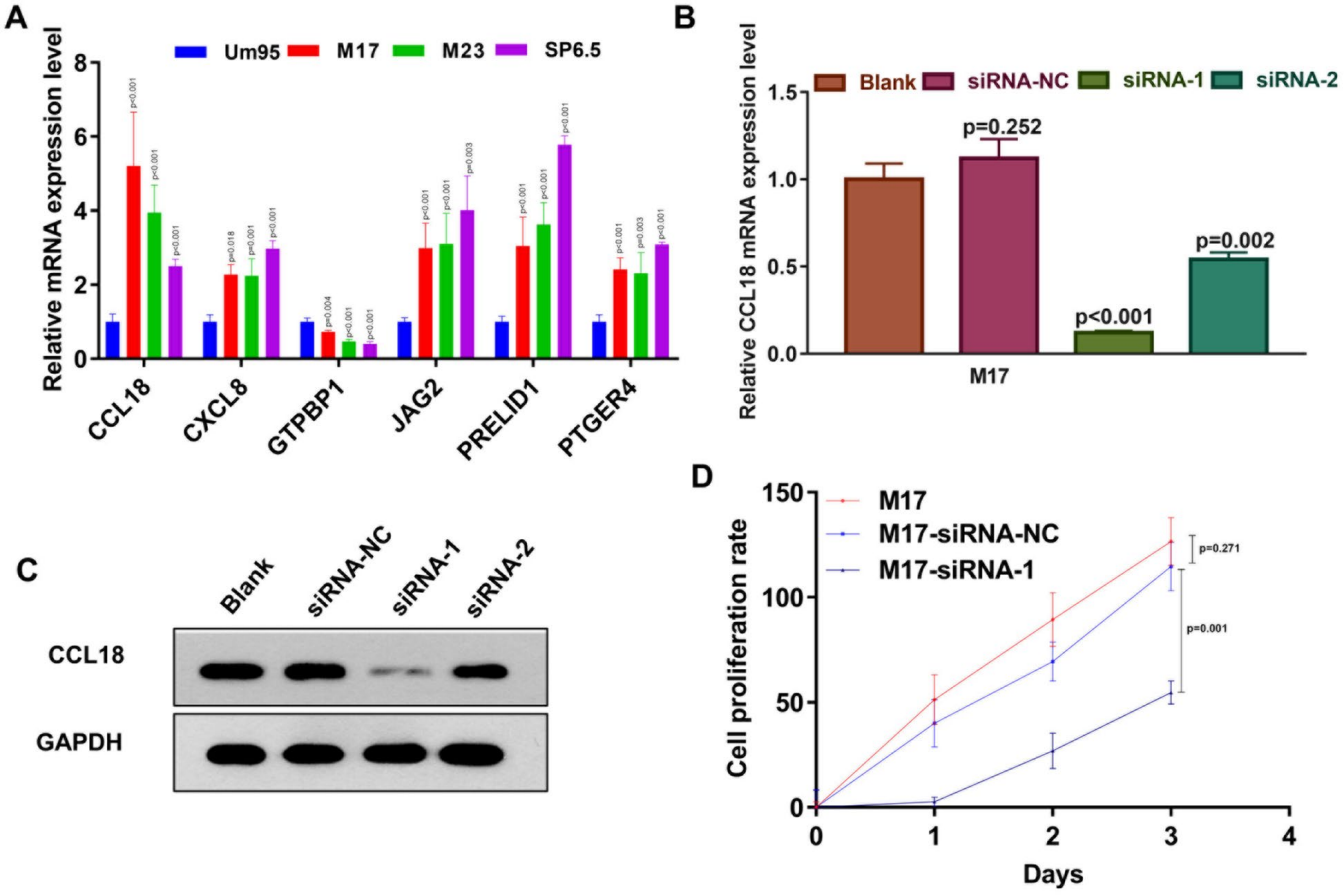
Knocking down of CCL18 significantly inhibits the proliferation of M17 cells. **A** qPCR experimental results of CCL18, CXCL8, GTPBP1, JAG2, PRELID1 and PTGER4 in Um95, M17, M23 and SP6.5 cells; (**B**) CCL18 mRNA expression level of M17 cells (blank) and siRNA-NC, siRNA-1 and siRNA-2 treated M17 cells; (**C**) CCL18 protein expression level of M17 cells (blank) and siRNA-NC, siRNA-1 and siRNA-2 treated M17 cells; **D**: CCK8 counting results of M17 cells (blank) and siRNA-NC and siRNA-1 treated M17 cells

**Fig. 11:**
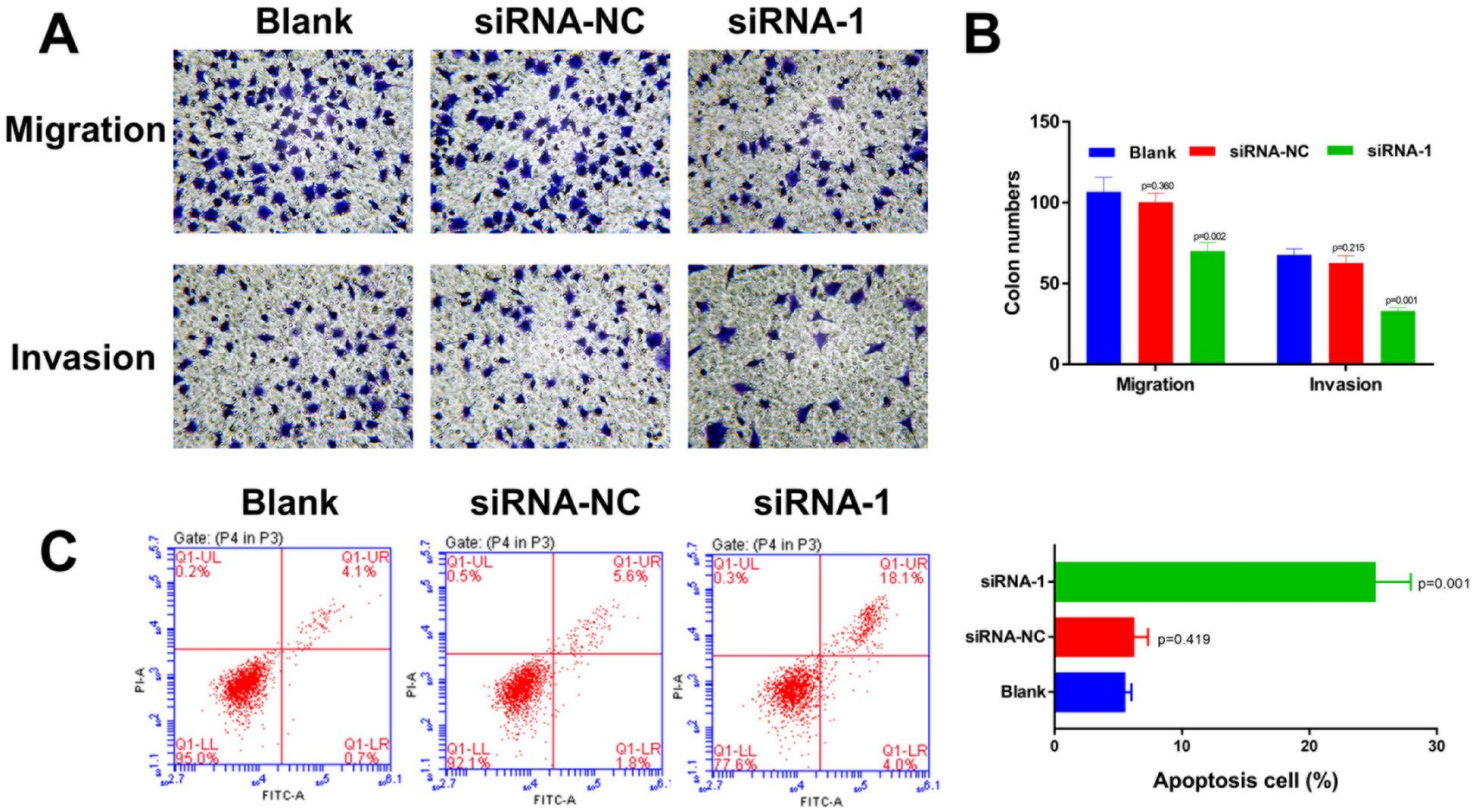
Knockdown of CCL18 inhibits M17 migration and invasion and induces M17 cell apoptosis. **A** Trans-well test results of M17 cells (blank) and siRNA-NC and siRNA-1 treated M17 cells migration (upper) and invasion (lower) capacity; **B** Data statistics of Fig A; **C** Flow cytometric cell apoptosis detection of M17 cells (blank) and siRNA-NC and siRNA-1 treated M17 cells

To verify our computational findings, we tested the mRNA expression level of CCL18, CXCL8, GTPBP1, JAG2, PRELID1 and PTGER4 in uveal melanoma cell lines: M17, M23 and SP6.5 and normal uveal epithelial cell: Um95. We found that compared with Um95, the expression patterns of these six genes in uveal melanoma cell lines: are consistent with our computational findings (Fig. 10A). In addition, we found that CCL18 has the highest expression in the M17 cell line, so we selected the CCL18 gene to perform related functional verification in M17 cells. As shown in Fig. 10B-C: siRNA-2 successfully knocked down the expression of CCL18 in M17 cells. The results of cell proliferation experiments show that low CCL18 expression can significantly inhibit the proliferation ability of M17 cells (Fig. 10D). Inhibition of CCL18 expression will significantly inhibit the migration and invasion of M17 cells (Fig. 11A-B). Furthermore, knocking down the expression of CCL18 significantly induced M17 cell apoptosis (Fig. 11C). In summary: our results demonstrated that our computational findings were consistent with the trend of in vitro cell experiments.

## Discussion

In this study, we identified 130 prognostic immune genes that strictly met the criteria of both univariate Cox regression analysis and K-M survival analysis, then used LASSO and multivariate Cox regression analyses to ultimately generate six immune-genes to construct an immune-related signature for predicting the prognosis of UVM patients. We found that the overall survival time was shorter of patients in the high-risk group than those in the low-risk group. Moreover, taken together with the results of time-dependent and multivariate ROC analysis, it showed a satisfactory diagnostic efficacy. In addition, the predictive independence was also confirmed. Most importantly, these findings were validated in two external GEO datasets.

Previous studies have reported some clinical variables that may affect the prognosis of UVM patients, including fair skin, light-colored eyes, congenital ocular melanocytosis, karnofsky index, largest dimension of the largest metastasis site, metastatic burden, serum transaminase, lactate dehydrogenase, and alkaline phosphatase level [2, 21, 22]. These clinical factors may really help clinical doctors offer optimal treatment and make a good prognosis prediction for UVM patients. However, these variables had not been recorded in the TCGA dataset. Therefore, instead, we included some typical variables in the dataset, such as the histological subtype, clinical stage, new tumor event, tumor basal diameter, tumor thickness, and some traditional demographical indexes, including age, and gender. Surprisingly, we came out that only age and our immune-related signature were found as independent risk factors. Age as an independent risk factor was also identified on a metastatic UVM research [23], but it was not consistent with another immune-related signature published study previously [24].

Compared with the previously published immune biomarker study by Li et.al [24], we did make some progresses on some aspects. First, half of these six-immune-genes we selected had been validated to be correlated with UVM functions in vitro, while none of the previous study. For example, JAG2 promoted UVM cells growth and metastasis [25]. Besides, inflammation-induced CXCL8 might stimulate the UVM cells chemotactic capacity [26]. CCL18 could enhance UVM cell line growth through coculture with human retinal pericytes [27]. In addition, the function of the left three immune genes were also cancer-related [28–30]. Second, in our study, the AUC values of time-dependent ROC were higher in both 1- and 3 year (0.962 *vs* 0.82, 0.962 *vs* 0.94, respectively), which demonstrated a better diagnostic efficacy. Moreover, we also performed a multivariate ROC analysis, which directly showed that our immune-related signature was better than any other clinical variables. Third, our newly identified immune-related signature was also successfully validated in two external GEO datasets, which suggested a potential clinical application. Fourth, and importantly, we first constructed an immune-related nomogram model combining multiple clinical variables, together with this signature risk score in the UVM dataset cohort. The result of calibration analyses demonstrated a good consistence between the predicted and actual curves. Taken together with the K-M survival analysis and ROC curves, this prognostic six-immune-gene signature can accurately predict the OS of UVM patients and exhibit great potential for clinical applications, including individualized prognosis and therapy.

To further confirm that the enrichment function of this signature is truly correlated with immune function, first, we did a functional enrichment analysis on the differently expressed genes between the high-risk and low-risk groups, and the result demonstrated an enrichment of immune-related pathways, including response to interferon gamma, T cell activation, interferon-gamma-mediated signaling pathway, cellular response to interferon-gamma, antigen processing and presentation of peptide antigen, which actually successfully supported our findings. Second, we also performed the ssGSEA to evaluate the enriched types of immune cells and functions in high- and low-risk group patients, and it came out that high risk patients were enriched of many immune cells, including B cells, CD8 + T cells, DCs, macrophages, pDCs, Tfh, Th2 cells, TIL, and Treg, while low risk patients only enriched in a DCs. Moreover, high risk patients were enriched of all immune functions, except for APC-co-inhibition and Type-II IFN response. Taken these two kinds of data together, we believed that this newly identified signature as a biomarker is useful to predict the prognosis on the immune therapy.

There are some limitations in our current study. First, these findings were obtained through mRNA level in public databases, so the following validations on protein expression level, in-vivo and clinical sample are needed. Second, external validation on patients treated with immunotherapy is also needed to further confirm the application of our signature and nomogram model. Therefore, we will continue to conduct an in-depth study to illustrate the molecular mechanisms of six immune genes, and make this signature and nomogram model more convincing for clinical application in the future.

## Conclusions

We successfully constructed a prognostic six-immune-gene signature using public TCGA UVM dataset and validated it in two GEO datasets. This signature was confirmed to have promising diagnostic and predictive efficacies as a biomarker. In addition, the novel nomogram model was confirmed as a good predictive biomarker. These findings could provide UVM patients with individualized clinical prognostic prediction and potential novel treatment targets.

## Supplementary Information

**Additional file 1.** The original, uncropped blots for WB.

## Data Availability

All data of this study are available in TCGA-UVM dataset (URL: https://portal.gdc.cancer.gov/) and two GEO datasets (URL: https://www.ncbi.nlm.nih.gov/gds/?term=GSE84976, https://www.ncbi.nlm.nih.gov/gds/?term=GSE22138).

## Abbreviations

TCGA: The Cancer Genome Atlas
GEO: Gene Expression Omnibus
UVM: Uveal melanoma
LASSO: Least absolute shrinkage and selection operator
GO: Gene Ontology
KEGG: Kyoto Encyclopedia of Genes and Genomes
ssGSEA: Single sample Gene Set Enrichment Analysis
BP: Biological process
CC: Cell component
MF: Molecular function
