# Supplementary figures and images for: Identification of a prognostic six-immune-gene signature and a nomogram model for uveal melanoma
Source: BMC Ophthalmol. 2023 Jan 3;23:2. doi: 10.1186/s12886-022-02723-1 (PMC9809105; doi:10.1186/s12886-022-02723-1)

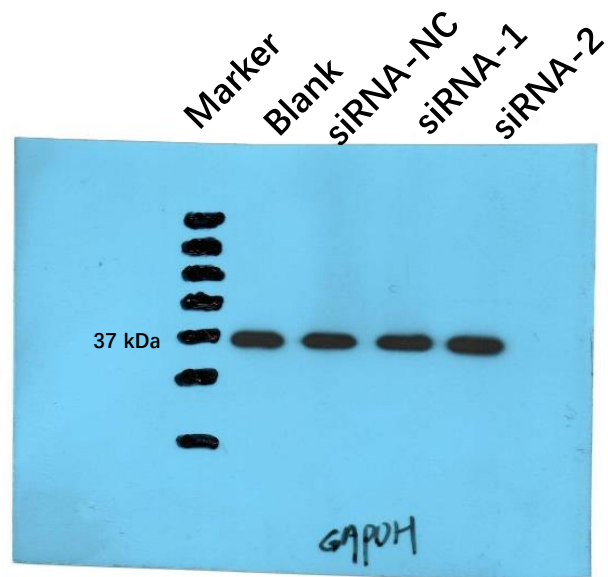

GAPDH

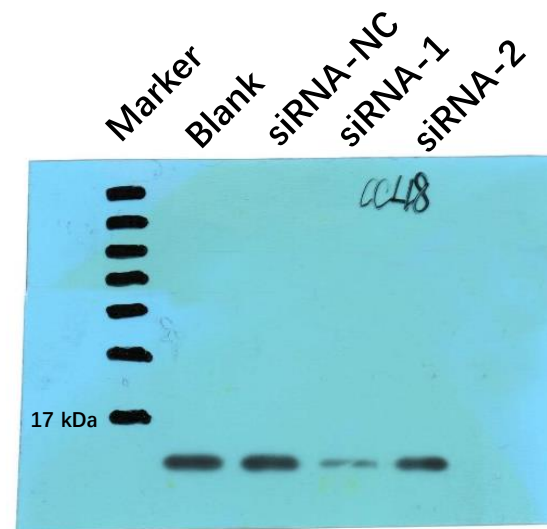

CCL18

The original, uncropped blots for WB

Supplement: Supplementary file 1 — Additional file 1. The original, uncropped blots for WB. [file 12886_2022_2723_MOESM1_ESM.pdf]
